# FEASIBILITY AND ACCEPTABILITY OF MBCT AND BA TO REDUCE PRE-DEATH GRIEF IN FAMILY CAREGIVERS OF PERSONS WITH DEMENTIA

**DOI:** 10.1093/geroni/igae098.4348

**Published:** 2024-12-31

**Authors:** Benjamin Neugebauer, Caroline Cummings, Sarah Sparks, Lauren Elliott, Volker Neugebauer, Lauren Chrzanowski, Elisabeth McLean, Jonathan Singer

**Affiliations:** Texas Tech University, Lubbock, Texas, United States; Texas Tech University, Lubbock, Texas, United States; Texas Tech University, Lubbock, Texas, United States; Texas Tech University, Lubbock, Texas, United States; Texas Tech University Health Sciences Center, Lubbock, Texas, United States; Texas Tech University, Lubbock, Texas, United States; Texas Tech University, Lubbock, Texas, United States; Texas Tech University, Lubbock, Texas, United States

## Abstract

Family caregivers of individuals with Alzheimer’s Disease and Alzheimer’s Disease Related Dementia (AD/ADRD) may experience pre-death grief (PDG). Behavioral activation (BA) and mindfulness-based cognitive therapy (MBCT) improve grief outcomes for bereaved individuals but neither have been investigated in the context of PDG. Notably, there are high rates of treatment attrition for family caregivers of persons with AD/ADRD underscoring the need to improve treatment options for this population. The present study is Stage I of NIH Stage Model for Behavioral Intervention Development that examines the acceptability and feasibility of two interventions. Participants in West Texas have limited to no resources due to a lack of respite, neurologists, and caregiving interventions, as they live 5 hours from a large urban setting. Family caregivers in West Texas were randomized to receive MBCT or BA following administration of neuropsychological and psychological assessments. Participants completed a shorter battery of assessments each session, and the full battery during the final session and at all follow ups. Thirty-three participants were recruited and consented, 84.8% (n=28) completed the study, 96.0% (n=27) of participants completed the final survey, and 86% (n=24) completed the 1-month follow up. When comparing the number of sessions attended by participants receiving MBCT vs. BA, there was no significant differences (p=0.73). Qualitatively, participants endorsed enjoying the interventions, and reported they were not burdensome. Results support the feasibility and acceptability of these evidence-based interventions for family members of persons with AD/ADRD in a unique West Texas sample.

